# Crystal structure of the magnetobacterial protein MtxA C-terminal domain reveals a new sequence-structure relationship

**DOI:** 10.3389/fmolb.2015.00025

**Published:** 2015-05-21

**Authors:** Geula Davidov, Frank D. Müller, Jens Baumgartner, Ronit Bitton, Damien Faivre, Dirk Schüler, Raz Zarivach

**Affiliations:** ^1^Department of Life Sciences and the National Institute for Biotechnology in the Negev, Ben-Gurion University of the NegevBeer Sheva, Israel; ^2^Department of Microbiology, University of BayreuthBayreuth, Germany; ^3^Department of Biomaterials, Max Planck Institute of Colloids and Interfaces (MPI)Potsdam, Germany; ^4^Department of Chemical Engineering, Ilse Katz Institute for Nanoscale Science and Technology, Ben Gurion University of the NegevBeer-Sheva, Israel

**Keywords:** MtxA, magnetotactic bacteria, magnetosome genomic island, magnetotaxis, magnetosome, tetratricopeptide repeats, immunoglobulin-like domain

## Abstract

Magnetotactic bacteria (MTB) are a diverse group of aquatic bacteria that have the magnetotaxis ability to align themselves along the geomagnetic field lines and to navigate to a microoxic zone at the bottom of chemically stratified natural water. This special navigation is the result of a unique linear assembly of a specialized organelle, the magnetosome, which contains a biomineralized magnetic nanocrystal enveloped by a cytoplasmic membrane. The *Magnetospirillum gryphiswaldense* MtxA protein (MGR_0208) was suggested to play a role in bacterial magnetotaxis due to its gene location in an operon together with putative signal transduction genes. Since no homology is found for MtxA, and to better understand the role and function of MtxA in MTBés magnetotaxis, we initiated structural and functional studies of MtxA via X-ray crystallography and deletion mutagenesis. Here, we present the crystal structure of the MtxA C-terminal domain and provide new insights into its sequence-structure relationship.

## Introduction

Magnetotactic bacteria (MTB) are a heterogeneous group of aquatic microorganisms that share the ability to orient themselves along geomagnetic field lines. This ability is achieved by unique organelles—magnetosomes—that are synthesized by the bacteria for supposedly passive orientation along the magnetic field. This organelle is characterized by its ability to grow one magnetic nanocrystal (greigite, Fe_3_S_4_ or magnetite, Fe_3_O_4_) per vesicle under ambient conditions (Faivre and Schüler, 2008).

The magnetosomes are arranged into single or multiple chain-like structures that enable the cell to dynamically align along external magnetic fields, a behavior known as magnetotaxis (Schüler, 2008; Lefevre et al., 2014; Zhu et al., 2014). Magnetotaxis, in combination with aerotaxis and perhaps phototaxis, is thought to direct the swimming of cells toward growth-favoring microoxic zones at the bottom of chemically stratified natural waters (Frankel et al., 1997; Frankel and Bazylinski, 2006; Bennet et al., 2014; Popp et al., 2014). Magnetosome formation, magnetite biomineralization as well as magnetosome organization are controlled by a large set of soluble and integral membrane proteins, most of which are unique, largely encoded by genes clustered within a genomic magnetosome island (MAI) (Schübbe et al., 2003; Komeili, 2007; Richter et al., 2007; Kolinko et al., 2014; Nudelman and Zarivach, 2014).

Previously in the alphaproteobacterium *Magnetospirillum gryphiswaldense*, the MtxA protein (MGR_0208), which is in or attached to the magnetosome membrane, was identified by a comparative genomic study as “MTB-specific.” This means that it is highly conserved in other MTB, but absent from non-magnetic bacteria (Richter et al., 2007). *mtxA* is encoded within a gene cluster located near the MAI that is also partly conserved among MTB (Richter et al., 2007). Orthologous proteins of MtxA are present in MTB strains *Magnetospirillum* sp. AMB-1, *Magnetospirillum magnetotacticum* strain MS-1 and *Magnetococcus marinus* MC-1. These proteins exhibit high amino acid sequence identity (60%, 60%, and 50%, respectively) and have identical lengths (Matsunaga et al., 2005). All MtxA proteins contain a predicted signal peptide sequence and some of these proteins have a structural element that forms repeat motifs that are defined by possessing duplications of a basic sequence motif usually involved in the formation of a structural element of the protein (Andrade et al., 2001). One of the protein repeats is a 34-amino acid motif called tetratricopeptide repeats (TPR); each repeat of the TPR domain forms a helix-turn-helix structure that serves as a template for protein-protein interactions that can mediate the assembly of multi-protein complexes. The number of observed TPR units in proteins ranges from 3 to 16, which are generally arranged as tandem arrays (Zeytuni and Zarivach, 2012). TPR structural elements have confirmed the general folding pattern with variations in structure, as well as adaptations that provide the TPR-containing proteins with alternative modes of interaction with different binding partners (D'Andrea and Regan, 2003; Zeytuni and Zarivach, 2012).

In its gene cluster, *mtxA* is followed by a highly conserved gene with similarities to Pfamés adenylate cyclase protein family model and CHASE2 domain in MTB strains. This domain organization belongs to a widespread group of transmembrane receptors that function as sensors for monitoring environmental changes and regulatory circuit function in catabolite repression in microorganisms. Major types of these sensor proteins share conserved intracellular domains (Zhulin et al., 2003). A few of the proteins that participate in intracellular interactions have an immunoglobulin-like (Ig-like) domain which has a rich, β-strand domain that forms an antiparallel, β-sandwich with a topology analogous to an Ig constant domain (Bazan, 1990). Ig-like domain proteins are also found in bacteria and these bacterial immunoglobulin-like (Big) domains are found in various varied functional proteins (Bateman et al., 1996).

Because of its co-localization with putative signal transduction proteins within the same operon, *mtxA* was suggested to play a role in magnetotaxis (Richter et al., 2007). Another hypothesis of MtxA function is that it could be involved in the biomineralization of magnetite inside the magnetosome vesicle (Tanaka et al., 2006). However, *in vitro* assays suggested that, despite the possibility that the protein can bind to magnetite, it will likely not affect the crystallization of the mineral *in vivo* (Baumgartner et al., 2014). Since no obvious homology to any known protein could be found on the sequence level, we initiated genetic and structural studies of MtxA in order to better understand its role and possible involvement in magnetotaxis via structural identification of active folds/structures and via *in vivo* studies of an *mtxA* deletion mutant. Here, we present the *in vivo* cellular and *in vitro* structural analysis of the MGR_0208 deletion mutant lacking the signal peptide (MtxA_Δ1−24_).

## Materials and methods

### Expression of the MtxA_Δ1−24_ gene in *escherichia coli*

The gene construct *mtxA_Δ1−24_* was amplified from *M. gryphiswaldense* MSR-1 genomic DNA by PCR (oligonucleotides produced by MWG Operon) using KOD polymerase (Novagen). The amplified *mtxA_Δ1−24_* gene was ligated between the KpnI and SacI restriction sites of the pET-51b(+)Ek/LIC vector (Novagen), giving rise to plasmid pET51bMtxA_Δ1−24_MSR1 (Baumgartner et al., 2014). In this construct, the *mtxA_Δ1−24_*gene was fused in-frame between an N-terminal Strep-tag and a C-terminal His-tag. MtxA_Δ1−24_ has a calculated mass of 34,774 Da.

*E. coli* BL21 strain cells harboring the MtxA_Δ1−24_MSR-1 plasmid were grown in LB medium containing ampicillin (50 mg/mL) at 37°C at 190 rpm.Isopropyl-d-thiogalactopyranoside (IPTG) was added to 0.5 mM when an optical density of 0.6 OD was measured at 600 nm, to induce protein expression for an additional 16 h at 20°C at 190 rpm. The cells were harvested by centrifugation at 7438 g for 10 min at 4°C.

Selenomethionine (SeMet) incorporation to MtxA_Δ1−24_ protein was carried out using the SeMet minimal medium (Guerrero et al., 2001). A 30 mL overnight culture was grown in LB medium at 190 rpm. The cells were harvested at 2629 g for 10 min and the cell pellet was used as an inoculum for 1 L of 1X M9 minimal medium supplemented with ampicillin (50 mg/mL) and other additives (Guerrero et al., 2001). 50 μg/mL l-selenomethionine, 100 μg/mL l-lysine, 100 μg/mL l-threonine, 100 μg/mL phenylalanine, 50 μg/mL l-leucine, 50 μg/mL l-valine, and 50 μg/mL l-proline were added when an optical density of 0.6 was measured at 600 nm. The culture was grown for 30 min before IPTG was added to induce protein expression for an additional 16 h at 20°C at 190 rpm. The cells were harvested by centrifugation at 7438 g for 10 min at 4°C.

### Purification of MtxA_Δ1−24_

MtxA_Δ1−24_-expressing cells were suspended in lysis buffer (20 mM Tris-HCl pH 8.5, 300 mM NaCl, 20 mM imidazole, 0.02% Triton X-100) and incubated with DNase I (10 mg/mL) and a protease inhibitor cocktail [100 mM phenylmethylsulfonyl fluoride (PMSF), 1.2 mg/mL leupeptin and 1 mM pepstatin A] at a ratio of 1:1000 with binding buffer for 20 min at 4°C. The cells were then disrupted by two cycles in a French press pressure cell at 207 MPa. Cell debris was separated by centrifugation at 19,000 g for 90 min at 4°C and the soluble fraction was applied onto a 5 mL bed-volume homemade gravity Ni-NTA column (2.5 cm diameter, Econo-Column Chromatography Columns, Bio-Rad. Ni-NTA His Bind Resin; Lot M0063428, Novagen) that was pre-equilibrated with lysis buffer.

The protein was washed with four different wash buffers, as it leads to the best purified protein without contaminations: I (20 mM Tris-HCl pH 8.5, 1 M NaCl, 25 mM imidazole); II (20 mM Tris-HCl pH 8.5, 700 mM NaCl, 30 mM imidazole); III (20 mM Tris-HCl pH 8.5, 500 mM NaCl, 40 mM imidazole); IV (20 mM Tris-HCl pH 8.5, 300 mM NaCl, 50 mM imidazole) and was eluted with elution buffer (20 mM Tris-HCl pH 8.5, 200 mM NaCl, and 500 mM imidazole). The eluted protein was dialyzed against ion-exchange (IEX) buffer A (20 mM Tris-HCl pH 8.5, 50 mM NaCl) for 16 h at 4°C, after which the protein was applied onto a column of MonoQ 4.6/100 PE (GE Healthcare Biosciences) equilibrated with IEX buffer A. The protein was eluted with a linear gradient of 50–2000 mM NaCl in IEX buffer B.

The relevant protein peak was collected and concentrated using a Vivaspin-4 10,000 mWCO (Sartorius Stedim Biotech GmbH) and applied onto a column of HiLoad 26/60 Superdex 200 (GE Healthcare Biosciences) equilibrated with size-exclusion chromatography (SEC) buffer (20 mM Tris-HCl pH 8.5, 200 mM NaCl). Purified MtxA_Δ1−24_ was then concentrated to 47 mg/mL for crystallization, flash frozen in liquid nitrogen and stored at −80°C. Sample purity at this stage was analyzed by SDS-PAGE and protein identification was confirmed by tandem mass spectroscopy.

A cell harvesting and purification protocol similar to that used for native MtxA_Δ1−24_ protein was carried out with the SeMet MtxA_Δ1−24_ protein. Purified SeMet MtxA_Δ1−24_ was then concentrated to 52 mg/mL for crystallization, flash frozen in liquid nitrogen and stored at −80°C. Sample purity at this stage was analyzed by SDS-PAGE and the mass spectra obtained from the native MtxA_Δ1−24_ protein and the SeMet MtxA_Δ1−24_ protein were compared for differences (data not shown).

### Circular dichroism of MtxA_Δ1−24_

Circular dichroism (CD) measurements were conducted with a J750 Spectropolarimeter (Jasco Inc, Maryés Court, Easton, USA) equipped with a Pelletier device. MtxA_Δ1−24_ protein sample was prediluted to 0.2 mg/mL in buffer containing 20 mM Tris-HCl pH 8.5, 200 mM NaCl and measured with a 0.1 cm optical path Suprasil quartz cuvette (Hellma GmbH & Co., Müllheim, Germany). Spectra profiles of the samples were measured at a wavelength range of 190–240 nm at ambient temperature with bandwidth set to 1 nm, scan speed set to 10 nm/min and a time constant of 4 s. The thermal denaturation experiments of MtxA_Δ1−24_ were conducted by monitoring the dichroic absorption at a wavelength of 222 nm as a function of increased temperature varying from 25°C to 75°C at a heating rate of 1.0°C × min^−1^. The thermodynamic parameters associated with the temperature-induced denaturation were obtained by nonlinear, least-squares fitting analysis of the temperature dependence of CD, and a two-state denaturation process was assumed for the curve fit.

### Small angle X-ray scattering (SAXS) data collection of MtxA_Δ1−24_

All protein samples for SAXS measurements were taken from the previously mentioned SEC purification to eliminate products of complex formation or aggregation. MtxA_Δ1−24_ was diluted with 20 mM Tris-HCl pH 8.5, 200 mM NaCl. SAXS measurements were performed at the French national synchrotron facility SOLEIL, on the SWING beamline. The incident beam energy was 12 keV. The sample-to-detector (Aviex CCD) distance was set to 1892 mm, covering a *q*-range of 0.004–0.7 Å^−1^. All experiments were temperature controlled at 25°C. Typically, 55 successive frames of 0.5 s each were recorded for the protein solution and its corresponding buffer. Each frame was first angularly averaged and the final spectrum and experimental error were obtained by averaging over all frames and subtracting the pure solvent spectrum from the sample spectrum. Intensities were scaled using the scattering of water (Carn et al., 2012).

### SAXS data analysis and envelope model of MtxA_Δ1−24_

The radius of gyration (*R*_g_) was evaluated using the Guinier approximation (Guinier and Fournet, 1955). The GNOM program was used to obtain the pair-distance distribution functions, the corresponding maximum dimension of protein complexes (*D*_max_) and to determine the value for *R*_g_ from the entire scattering profile (Svergun, 1992). *Ab initio* envelopes were generated by the program DAMMIN (Svergun, 1999) using atomic radii set to the dummy atom packing radius determined by DAMMIN without imposing symmetry operation. Five DAMMIN runs were performed for every sample and an averaged dummy ball model (DBM) was generated by DAMAVER (Volkov and Svergun, 2003). The generated envelope models (DBMs) were fit using the Coot software (Emsley and Cowtan, 2004) and visualized using PyMOL (DeLano, 2002).

### Crystallization of MtxA_Δ1−24_

MtxA_Δ1−24_ was crystallized using the sitting-drop vapor-diffusion method at 20°C. 0.5 μL of MtxA_Δ1−24_ and 0.5 μL reservoir solutions were mixed to form the drop. The first crystallization trials were performed with MtxA_Δ1−24_ concentrated to 12, 15, 20, and 30 mg/mL at 20°C and 13°C in 20 mM Tris-HCl pH 8.5, 200 mM NaCl (Zeytuni et al., 2012). We traced these plates for up to 3 months without any positive crystal hit. The lack of protein crystals could be the result of the long, flexible Strep-tag and, as such, several options were devised to overcome the crystallization problems, such as the removal of the tag by cloning or the use of protease to remove the tag while crystallizing the protein. While trying this approach, we performed crystallization trials with 15 mg/mL MtxA_Δ1−24_ incubated with trypsin protease (trypsin from bovine pancreas, T8003 SIGMA) at 20°C in 20 mM Tris-HCl pH 8.5, 200 mM NaCl, at a molar ratio of 1:1000 or 1:4000 (trypsin protease:MtxA_Δ1−24_) in the drop. The initial crystallization conditions were examined using commercial screening kits from Hampton Research (Index, Crystal Screen I + II Screens), Molecular Dimensions (Structure screen I + II HT) and Rigaku (Wizard I + II screens). Crystal hits were observed in different conditions in the Index screen (Hampton Research) and Wizard screen (Rigaku). The same protocol of crystallization trial with trypsin protease was performed for the SeMet MtxA_Δ1−24_ protein. Crystallization of the SeMet MtxA_Δ1−24_ protein was with the Index commercial screening kit (Hampton Research). Crystal hits were noticed in different conditions in the Index screen; condition No. 87 produced the same clusters of tiny needle crystals that were obtained using a reservoir containing 0.2 M sodium malonate pH 7.0 and 20% PEG 3350 after 24 h incubation. We then harvested the SeMet MtxA_Δ1−24_ crystals directly from the screen and flash froze them in liquid nitrogen without any cryo-soaking, due to the high concentration of the PEG 3350 that acted as cryo-protectant. One of the SeMet MtxA_Δ1−24_ crystals in the condition No. 87 diffracted to 2 Å resolution.

### Diffraction data collection and structure determination of MtxA_Δ1−24_

The native and SeMet Crystals were harvested and flash-cooled in liquid nitrogen without addition of a cryoprotecting solution. Diffraction data for native MtxA_Δ1−24_ were collected on beamline ID23-2 at the European Synchrotron Radiation Facility (ESRF, Grenoble, France), resulted in low resolution diffraction (data not showed). Diffraction data for the SeMet MtxA_Δ1−24_ protein were collected on beamline ID14-4 at the ESRF, which is equipped with an ADSC Q315r mosaic CCD detector. Data collection was performed at 100 K. For the SeMet MtxA_Δ1−24_ crystal form we scanned the crystal for existence of Selenium signal and to obtained f′ and f″ values (–8.60, 6.92 respectively). A peak data set was measured at a wavelength of 0.98 Å for a total of 267 frames with an oscillation range of 1° and an exposure time of 0.224 s per image. The crystal-to-detector distance was 347.46 mm. Data reduction and scaling were performed with HKL-2000 (Otwinowski and Minor, 1997). This data was used for Se-SAD phase calculation. Phases were obtained by the automatic data processing which has been available and in use on all Joint Structural Biology Group (JSBG) MX beamlines (Monaco et al., 2013). Phase statistics are: correlation coefficient CC(E) = 0.399, figure of merit 0.7551_acentric_/0.09483_centric_ and phasing power is 1.514. The output map and protein sequence were entered into the auto-build in ARP/wARP web service; http://www.embl-hamburg.de/ARP/ (Langer et al., 2008). The program placed correctly 230 out of the 650 residues and water molecules were added manually. The final model was built by Coot and refined in REFMAC (Vagin et al., 2004; Emsley et al., 2010). Structural figures were prepared with PyMOL (DeLano, 2002).

### *mtxA* deletion

A markerless in-frame deletion of *mtxA* (Mgr_0208) was carried out essentially as described (Raschdorf et al., 2014). Briefly, 1 kbp up- and downstream (including the first and the last four codons) of *mtxA* were amplified from genomic DNA, purified and fused by overlap-extension PCR. The resulting 2 kbp DNA fragment was purified, digested with BamHI and PstI, ligated into pORFM GalK blu and used to transform competent *E. coli* DH5α cells. Kanamycin resistant, white colonies were selected to isolate the resulting vector pIM01 and DNA sequencing was performed to verify absence of mutations within the homologous regions. Subsequently, the vector was transformed into *E. coli* BW29427. Transformation of *M. gryphiswaldense* with pIM01 was carried out by biparental conjugation as described (Schultheiss and Schüler, 2003; Ullrich and Schüler, 2010). Recombinant, merodiploid clones were isolated from kanamycin-supplemented modified flask standard medium (FSM) plates and screened for up or downstream integration of the plasmid. Counterselection was then performed on FSM plates supplemented with 0.5% (w/v) galactose. Successful deletion of *mtxA* was confirmed by PCR-screening with oligonucleotide primers specific to regions adjacent to the cloned homologous sections.

### Phenotypic characterization of the *mtxA* mutant

Swarm assays were carried out on swim agar plates as described (Popp et al., 2014). For transmission electron microscopy (TEM) imaging cells were grown at 25°C under microaerobic conditions to an OD_565_ of 0.1, fixed in formaldehyde (1%), concentrated, adsorbed onto carbon-coated copper mesh grids, and washed three times with particle-free water. Samples were viewed and recorded with a Morgagni 268 microscope (FEI, Eindhoven, the Netherlands) at an 80-kV accelerating tension. Magnetic responses (*C*_mag_) of exponentially growing *M. gryphiswaldense* cultures were measured photometrically at 565 nm as reported previously (Schüler et al., 1995).

## Results

### Protein sequence analysis of the putative magnetotaxis protein MtxA

**Figure 1:**
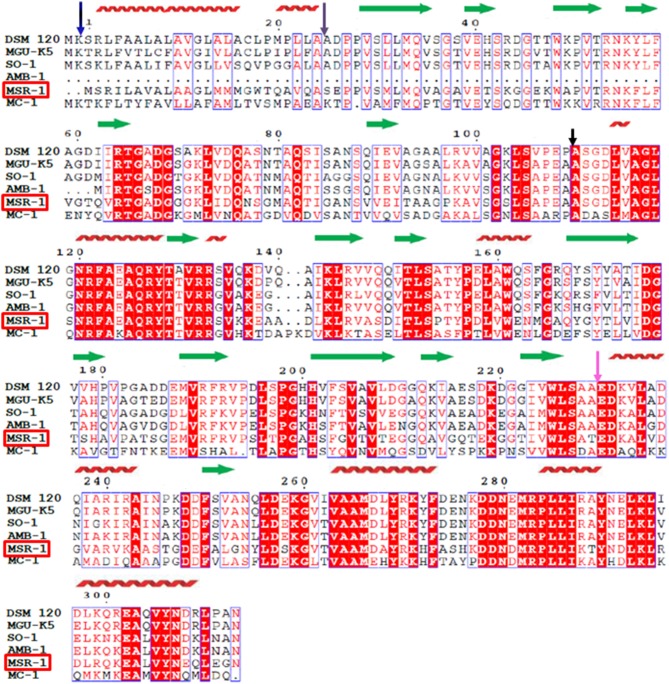
**Sequence alignment of magnetotaxis protein MtxA (MSR-1)**. Amino acid sequence alignment of the *Magnetospirillum gryphiswaldense* MSR-1 magnetotaxis protein MtxA MGR_0208 (MSR-1) with the *Phaeospirillum molischianum* DSM 120 PHAMO_340145 (DSM 120), the *Phaeospirillum fulvum* MGU-K5 K678_00055 (MGU–K5), the *Magnetospirillum* sp SO-1 H261_01492 (SO-1), the *Magnetospirillum magneticum* AMB-1 amb2230 (AMB-1) and the *Magnetococcus* sp. MC-1 Mmc1_3696 (MC-1). Strictly conserved residues are highlighted with a red background and highly homologous residues are boxed. The secondary structural elements of MSR-1 defined by the analysis of the structure by using the PSIPRED server are indicated as red coils for α-helices and green arrows for β-strands. The purple arrow indicates for the end of the signal peptide.

In our first attempt to characterize MtxA, we ran a multiple sequence alignment with different servers. In Figure 1, we present the output from the ClustalW2 server; http://www.ebi.ac.uk/Tools/msa/clustalw2 (Larkin et al., 2007) and ESPript 3.0; http://espript.ibcp.fr/ESPript/cgi-bin/ESPript.cgi (Gouet et al., 2003). Similarity search analyses revealed high homology at the amino acid sequence level in five different organisms, of which two are not MTB. All *mtxA* genes in all identified organisms represent uncharacterized proteins with highly conserved residues. The sequence comparison highlights that MtxA shares 61% identity with the *M. magneticum* AMB-1 amb2230 protein, 58% identity with *Magnetospirillum* sp. SO-1 H261_01492 protein and *Phaeospirillum molischianum* DSM 120 PHAMO_340145 protein, 57% identity with *Phaeospirillum fulvum* MGU-K5 K678_00055 protein, and 51% identity with *M*. sp. MC-1 Mmc1_3696 protein (Figure 1). All the other servers used (http://www.uniprot.org/align, http://blast.ncbi.nlm.nih.gov/Blast.cgi, http://www.st-va.ncbi.nlm.nih.gov/tools/cobalt) yielded similar results.

To further analyze the protein sequence, we ran secondary-structure prediction on the PSIPRED server; http://bioinf.cs.ucl.ac.uk/psipred (Buchan et al., 2010) (Figure 1). The secondary structure prediction indicated that MtxA has four different structural regions. The first 24 a.a. (blue arrow) that were predicted to fold mainly as an α-helix were previously recognized as a signal peptide sequence (Richter et al., 2007). The second region (25-110 a.a., purple arrow) is mainly predicted to be a β-strand region. The third region (110-230 a.a., black arrow) is predicted to fold as alternating α-helices and β-strands. The last region (231-313 a.a., pink arrow) is predicted to fold mainly as α-helices. The prediction did not find any transmembrane domain or long unstructured regions (Figure 1). We also ran a sequence comparison of MtxA against the Protein Data Bank (PDB), which shows only sequence similarities to very small segments within the MtxA sequence, with ~24–35% sequence identity (data not shown).

### Phenotype of the *mtxA* deletion mutant

Characterization of the *mtxA* mutant under standard culture conditions revealed a phenotype virtually indistinguishable from the wild type (wt). TEM analysis indicated that the mutant cells formed regular, wt-sized and -shaped crystals that are aligned in wt-like chains (Supporting Figure 6). Consistently, the mutant strain exhibited a wt-like magnetic response (not shown). Microscopic inspection showed that the mutant cells were motile, and a swarm assay on swim agar plates exposed to a magnetic field revealed that *mtxA* mutant cells aligned and moved parallel to the field as the wt, as indicated by distortion of the aerotactic swim ring in semisolid agar (Popp et al., 2014) (Supporting Figure 7). However, although we were unable to discern phenotypic differences between the wt and the *mtxA* mutant under the common standard growth conditions, this does not entirely preclude that the *mtxA* deletion might cause more subtle effects under some untested conditions.

### Characterization of MtxA_Δ1−24_

**Figure 2:**
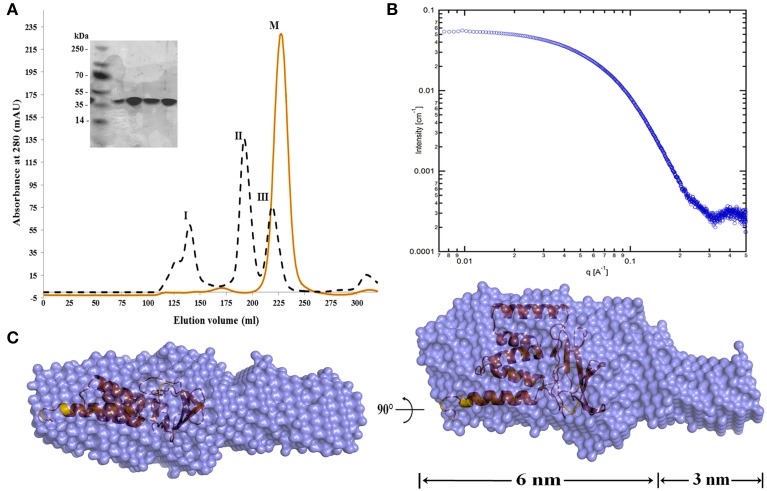
**MtxA_Δ1−24_ size-exclusion chromatography and SAXS envelope model. (A)** Size exclusion (Superdex 200 HiLoad 26/60) chromatography analysis of the MtxA_Δ1−24_ (red) and chromatographic separation and calibration curve for the standard proteins (black). The standard proteins are: I: Thyroglobulin (Mr = 669,000), II: Aldolase (Mr = 158,000) and III: Ovalbumin (Mr = 44,000) (Gel Filtration Calibration Kit HMW code no. 28-4038-42, GE). The elution volume of MtxA_Δ1−24_ (M) is at ~210 mL. Comparison with elution volumes of the standard proteins suggests that the protein is present as a monomer in solution (the molecular weight of MtxA_Δ1−24_ is 34.77 kDa). The eluted protein was separated by 15% SDS-PAGE gel and stained with Coomassie blue. **(B)** Small-angle X-ray scattering data of MtxA_Δ1−24_ in solution shown as individual data points. **(C)** The *ab initio* bead model of MtxA_Δ1−24_ calculated using DAMMAVER with the MtxA structure inside. Dummy atoms are shown as blue spheres, surrounded by a molecular surface and MtxA (orange) shown in cartoon (a.u., arbitrary unit).

To study the structure-function of MtxA and to avoid the secretion of MtxA to the periplasm, we sub-cloned a deletion mutant that includes Strep- and His-tags for detection and purification (MtxA_Δ1−24_). MtxA_Δ1−24_ was expressed as a soluble protein. For protein purification, we started with Ni-NTA affinity chromatography followed by ion exchange (MonoQ) and SEC (Superdex 200) (Figure 2A). Comparing MtxA_Δ1−24_ to the molecular size marker in the size-exclusion chromatogram indicates that MtxA_Δ1−24_ eluted as a monomer. SDS-PAGE analysis of the corresponding size-exclusion peak showed that the protein was highly purified and stable (Figure 2A).

To compare the predicted secondary structure elements with MtxA_Δ1−24_, we measured its (CD) curve at room temperature (Supporting Figure 1A). The observed double-minimum at 205 nm and 225 nm suggests contributions of β-strand (40.6%) and α-helical (3.4%) conformations, as estimated by the K2D3 analysis algorithm (Louis-Jeune et al., 2012). This value corresponds with our secondary structure prediction for a higher percentage of β-strand content. We have also analyzed the thermal stability of MtxA_Δ1−24_ by measuring the CD temperature dependence (25–75°C) at 222 nm (Supporting Figure 1B). The sigmoidal melting curve with an extrapolated melting temperature (Tm) of 50 ± 2°C further supports our assumption that MtxA_Δ1−24_ is a stable and soluble protein.

To further characterize the monomeric form of MtxA_Δ1−24_ in solution, we used small angle X-ray scattering (SAXS). The scattering data was collected at three different protein concentrations of 0.5, 1, and 2 mg/mL (Figure 2B). At these concentrations the scatterings are quite similar, indicating that the molecular dimensions are independent of protein concentration over this concentration range. To obtain a three dimensional model of MtxA_Δ1−24_, we generated a dummy-ball model (DBM) from the 2 mg/mL SAXS data. The model displays an elongated structure divided into large and small connected ellipsoids (Figure 2C). The long ellipsoid axes dimensions of the large and small ellipsoids are 6 and 3 nm, respectively.

To obtain a high resolution structure, we started with crystallization trials that were performed as described in M&M for native and SeMet MtxA_Δ1−24_(Supporting Table 1 for the crystal hits). Native crystals did not diffracted beyond 2.8 Å so we focused on crystal hits from the Index condition No.87 that had the largest non-multilayer distinct well faceted SeMet crystals. We harvested the SeMet MtxA_Δ1−24_crystals directly from the screen and flash froze them in liquid nitrogen without any cryo-soaking, due to the high concentration of the PEG 3350 that acted as cryo-protectant.

One of the SeMet MtxA_Δ1−24_ crystals diffracted to 2 Å resolution. Analysis of the diffraction data fulfilled the systematic absence rules of space group P2_1_2_1_2_1_. The unit cell parameters are *a* = 40.34 Å, *b* = 88.95 Å, *c* = 95.40 Å (Table 1). The crystal unit cell and space group parameters are equal to the parameters that had been obtained from the native MtxA_Δ1−24_ (data not shown). Since protease was used as part of the crystallization, MtxA_Δ1−24_ crystals are composed of some of the protein domains that result from the cleavage. The cloned MtxA_Δ1−24_ protein consists of 325 amino acids with an N-terminal Strep-tag and a C-terminal His-tag, whereas the full MtxA has 313 amino acids. Mass spectrometry analysis of the trypsin treated MtxA_Δ1−24_ exhibits a high intensity peak around 20.52 kDa and a less intense peak around 41.31 kDa that fits the MtxA_Δ1−141_ 20.67 kDa size (Supporting Figure 2). Furthermore, the residues seen in the electron density maps are L136-S311 (L142-Q313, MtxA_Δ1−141_). The Matthewsé coefficient calculation that was performed based on the unit cell parameters and the molecular weight for the L136-S311 residues is with a probability of 98%, assuming the presence of two monomers per asymmetric unit and a solvent content of 45.3%. Both are within the normal range of values observed for soluble protein crystals (Matthews, 1968; Winn et al., 2011).

**Table 1:** **Data collection and refinement statistics of SeMet MtxA_Δ1−142_**.

	**SeMet MtxA_Δ1−24_**
Data collection	ID14-4 – ESRF
Space group	P2_1_2_1_2_1_
**UNIT CELL PARAMETERS**	
a, b, c (Å)	40.34, 88.95, 95.40
α, β, γ (°)	90, 90, 90
Resolution (Å)	47.70–2.03 (2.07–2.03)
R_merge_ (%)	14.3 (65.9)
I/σ I	44.3 (3.6)
Completeness (%)	98.3 (91.3)
Redundancy	11.7 (4.9)
Wavelength (Å)	0.980
**REFINEMENT**	
Resolution (Å)	2.02
No. reflections (unique)	21827
*R_work_/R_free_*	18.48/22.7
**NO. ATOMS (NON HYDROGENS)**	
Protein	2800
Water	140
***B-FACTORS (Å^2^)***	
Protein (chain)	(A) −29.7, (B) −57.2
Water	33.7
***R.M.S. DEVIATIONS***	
Bond lengths (Å)	0.0179
Bond angles (°)	1.8023
Ramachandran statistics (%)	A: 97.6, B: 2.08, D: 0.3
*R_anomalous_*	0.68 (0.975)
PDB code	4Z29

*Values in parentheses are for the highest resolution shell. One crystal was used per data set. Data was collected at 100 K*.

**R*_merge_ = Σ_hkl_ Σ_i_|Ii(hkl) - <I(hkl)> |/Σ_hkl_ Σ_i_Ii(hkl), where Ii(hkl) is the observed intensity of an individual reflection and <I(hkl)> is the mean intensity of that reflection. 5% of the data were kept aside for the *R*_free_ calculation*.

*A, Fully allowed region, P, Partially allowed region and D, Disallowed region*.

### The overall fold of MtxA_Δ1−141_

**Figure 3:**
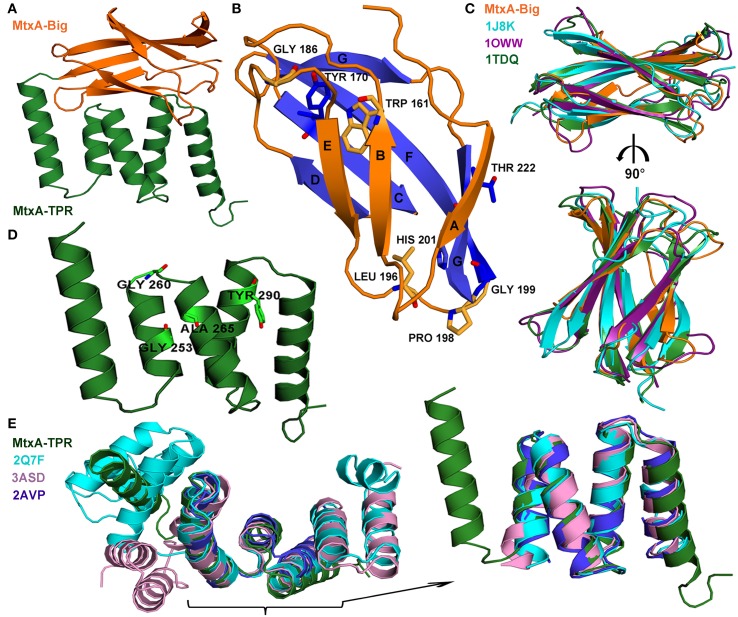
**MtxA_Δ1−141_ crystal packing and overall structures. (A)** The representative crystal packing of MtxA_Δ1−141_ structure contains a two-domain architecture: MtxA_142−226_ (MtxA-Big) and MtxA_227−313_ (MtxA-TPR) motifs. **(B)** The overall β-sandwich fold of the MtxA-Big structure; the seven β-sheets are labeled (A–F). The conserved amino acids are in stick representation. **(C)** Superimposition overlay of MtxA-Big with three of the Dali serverés alignments in cartoon representation reveals the high structural similarity: MtxA-Big in orange, 1J8K in cyan, 1OWW in deep purple and 1TDQ in forest. The molecules are shown in a 90° rotation view. **(D)** The overall fold of the (MtxA-TPR) structure contains two sequential TPR motifs. The conserved amino acids are represented as sticks. **(E)** Superimposition overlay of MtxA-TPR with some of the Dali server-aligned structures in cartoon representation reveals the high structural similarity: MtxA-TPR in green, 2Q7F in cyan, 3ASD in pink and 2AVP in blue, with enlargement of the high structural similarity area. All the structural images were prepared using PyMOL.

The crystal structure of MtxA_Δ1−141_ was determined by the automatic data processing server on beamline ID14-4 and auto-built with ARP/wARP and Coot. The structure was refined to an R factor of 18.5 and R_free_ of 22.7% (Table 1) and was deposited to the PDB (4Z29) (map quality is shown in Supplementary Figure 8). The overall structure of MtxA_Δ1−141_ has two distinguished domain architectures (Figure 3A): I- Ig-like fold (MtxA-Big) in orange, and II- TPR fold (MtxA-TPR) in green. The overall structure of MtxA_Δ1−141_ was back-fitted on the generated DBM from the SAXS data (Figure 2C). The structure displays a good fit to the longest ellipsoid structure.

Electrostatic surface characteristics of the MtxA_Δ1−141_ show several electrostatic charged patches and a main hydrophobic patch (Supporting Figure 4). The protein exhibits a highly negatively electrostatic potential charge at the bottom of the C-terminal MtxA-TPR domain (left, bottom). The hydrophobic patch covers the whole face created by the TPR and the Ig-like domains (top left, Supporting Figure 4).

The MtxA_Δ1−141_ structure displays two protein monomers in the asymmetric unit (Supporting Figure 5). The two monomers tightly contact each other with a buried surface area of 418 Å^2^. The interface between the two monomers is stabilized by a polar interaction network that involves hydrogen bonds, and charged and hydrophobic interactions (Supporting Figure 5). The hydrophobic interactions between the two monomers are comprised of highly conserved residues in MtxA, residues 282-287 “MRPLLI” (Figure 1), located at the Surface of the TPR domain. The surface around this position has a negative electrostatic charge (Supporting Figure 4).

### The fold of MtxA-Big and MtxA-TPR in MtxA_Δ1−141_ structure

The first domain—MtxA-Big: L142-L226 (MtxA_142−226_)—has a common Ig-like fold consisting of seven β-strands forming two antiparallel β-sheets, which pack against each other forming a β-sandwich (Figure 3B). In our structure monomer B, MtxA-Big domain has a higher B-factor than monomer A (37.4/20.3 Å^2^ respectively, calculated using all atoms, Supporting Figure 5B). Based on the crystal structure, monomer B has less surface area at the crystal contacts than monomer A, which may lead to a higher B-factor due to thermal instability. Alternatively, the higher B-factor may indicate for the MtxA-Big domain flexibility, which is part of the protein function. To see whether the MtxA-Big domain has similarities to other structures with similar topology available in the PDB, we used the Dali server; http://ekhidna.biocenter.helsinki.fi/dali_server/ (Dietmann et al., 2001). One of the best-generated fits with the MtxA-Big domain is fibronectin EDA (PDB ID code 1J8K, Z score = 8.4). Fibronectin type III (Fn-3) structures is a ~90-amino acid domain that forms seven β-strands in conserved regions of the chain (Niimi et al., 2001). These strands are folded into antiparallel β-sandwiches with a topology that is similar to immunoglobulin constant domains (Bazan, 1990). The MtxA-Big domain forms a β-sandwich fold with four β-strands on one side and three on the other side with no disulfide bonds, similar to some members of the Fn-3 family (Potts and Campbell, 1994). The first β-sheet (orange in Figure 3B) comprises strands A, B and E, whereas the second β-sheet (blue in Figure 3B) comprises strands C, D, F, and G. We overlapped MtxA-Big domain with three of the Dali matches: Tenascin-R (PDB ID code 1TDQ-A, Z score = 8.3) (Lundell et al., 2004), fibronectin EDA (PDB ID code 1J8K, Z score = 8.4) (Niimi et al., 2001) and Fibronectin first type III module (PDB ID code 1OWW, Z score = 7.4) (Gao et al., 2003) (Figure 3C). The structural superimposition shows that all adopt the same β-sheet arrangements, topology and conformations with RMS deviation of 1.4–1.57 Å, despite their low sequence identity (Supporting Table 2). Since the sequence identity is very low, we ran multiple sequence alignment in the ClustalW2 server to disclose significant highly conserved residues in Fn-3 structures (Supporting Figure 3A). The sequence alignment brought to light that MtxA-Big has the same highly conserved residues as in Fn-3 members: W161 (in β-sheet B), Y170 (in β-sheet C), and L196 (located in a loop between β-sheet E and β-sheet F) (Hoxha and Campion, 2014). We also found that P198 and G199 residues are highly conserved, as in some of the Fn-3 family members that are part of a structural conformation called the “tyrosine corner” (Hemmingsen et al., 1994). The “tyrosine corner” is a consensus sequence in Fn-3members: L-X-X-G-X-X-Y. MtxA-Big has only partial part of the consensus, L196, P198, G199 residues are conserved, but MtxA-Big lacks the sixth amino acid and instead of the tyrosine it has a histidine residue. In Fn-3 members, the Y and the L are close to each other (β-sheet E through β-sheet F) and the Y forms hydrogen bonds with a second a.a. in the consensus sequence above. The conserved H201 in MtxA-Big forms a hydrogen bond with a second a.a. (T197) and a hydrogen bond with the conserved D175 (Figure 1). This interaction probably preserves the β-sheet sandwich scaffold since MtxA-Big has shorter β-sheets C and D. Looking at the sequence alignment of MtxA, it discloses that there are another two residues that are well conserved: G186 and T222. G186 is in a loop between β-sheet D and β-sheet E and T222 at the C-terminal of the domain (β-sheets G).

The second domain, MtxA-TPR S227-N313 (MtxA_227−313_), contains five anti-parallel α-helices-and-turn motifs folded as tetra-trico-peptide repeat (TPR) motifs (Figure 3D). Again, we used Dali server for a structural superposition comparing this domain with proteins at the PDB. One of the best-generated fits with the MtxA-TPR domain is MamAR50E (PDB ID code 3ASD-A, Z score = 10.4) (Zeytuni et al., 2011). The TPR is a structural motif based on consensus sequence defined by a pattern of small and large hydrophobic amino acids, that adopts a helix-turn-helix fold that creates the repeating antiparallel α-helices (Zeytuni and Zarivach, 2012). Superimposition of MtxA-TPR domain structure with three Dali matched structures—synthetic consensus TPR protein (PDB ID code 2AVP-A, Z score = 10.4) (Kajander et al., 2007), MamAR50E (PDB ID code 3ASD-A, Z score = 10.4) (Zeytuni et al., 2011), and YrrB protein (PDB ID code 2Q7F-B, Z score = 11.3) (Han et al., 2007)—shows that they all adopt exactly the same TPR fold (Figure 3E) but share low sequence identity (Supporting Table 2). The structural superimposition of MtxA-TPR with 2AVP-A, 3ASD-A and 2Q7F-B results with RMS deviation of 1.2–1.39 Å with similar conformations of helix-turn-helix (Figure 3E and Supporting Table 2). We ran multiple sequence alignment in ClustalW2 server (Supporting Figure 3B) to search for the known conserved residues in the MtxA-TPR motifs because the result from the TPRpred server; http://toolkit.tuebingen.mpg.de/tprpred (Biegert et al., 2006) determined that there are no significant repeats in MtxA. The MtxA-TPR sequence alignment indicates that some of the consensuses pattern of conserved residues exists: G253 (position 8 in the N-terminal motif), G260 (position 15 in the N-terminal motif), A265 (position 20 in the N-terminal motif), and Y290 (position 11 in the N-terminal motif of the second repeat). The MtxA-TPR also keeps the pattern of hydrophobic amino acids, although it has variations in the consensus positions that preserve the motif of a helix-turn-helix (D'Andrea and Regan, 2003). Furthermore, one of these changes is a highly conserved residue in all the MTB strains. The difference in the consensus P32 position (between TPR motifs) (Zeytuni and Zarivach, 2012) is K277 in the MtxA-TPR. This K277 keeps the highly conserved sequence of “K-D-D-N” that holds the TPR-turn in MtxA.

**Figure 4:**
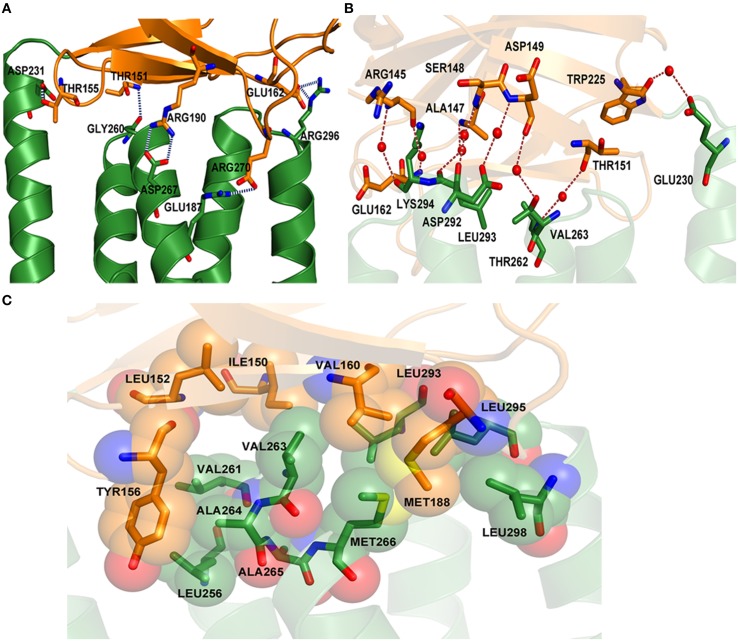
**Polar and hydrophobic interaction network of MtxA-Big with MtxA-TPR. (A)** “Close up” view of the hydrogen-bond interaction network (blue dotted lines) between the residues from each fold backbone: T151, T155, E162, E187, and R190 (MtxA-Big; orange cartoon; side chains shown as sticks) with D231, G260, D267, R270, and R296 (MtxA-TPR; green cartoon; side chains shown as sticks). **(B)** Overview of water-mediated interactions (red dotted lines) between the side chains (shown as sticks) and water (red spheres): R145 with K294, R145 with E162, A147 with L293, S148 with D292, D149 with D292, D149 with T262, T151 with V263, and W225 with E230. **(C)** Surface representation of hydrophobic interacting residues (shown as sticks and surface).

MtxA-Big and MtxA-TPR contact each other extensively in a parallel orientation with a buried surface of 558 Å^2^. The interface between the two folds is stabilized by a polar and hydrophobic interaction network (Figure 4). The polar network involves polar, hydrophobic and charge interactions. The hydrogen bonds are: T151 with G260, T155 with D231, E162 with R296, E187 with R270, and R190 with D267 (Figure 4A). The amino acids that interact with water molecule and form hydrogen bond network are: R145, A147, S148, D149, T151, E162, W225, E230, T262, V263, D292, L293, and K294 (Figure 4B). The hydrophobic interaction network is comprised of three main regions (Figure 4C). The first region is composed of hydrophobic interactions of Y156 with the side chains of V261, A264, and L256. Another hydrophobic interactionsé network is the side chain of I150 with the side chain of V263, V160, and L293. The last network embraces hydrophobic interactions between the side chain of V160, M188, A265, M266, L295, and L298.

## Discussion

In this study, we present the analysis of MtxA, a protein that has been suggested to play a role in magnetotaxis (Schüler, 2008; Zhu et al., 2014). However, considering the lack of a phenotype of the *mtxA* deletion mutant we conclude that MtxA is essential for magneto-aerotaxis in *M. gryphiswaldense*, although it might have more subtle functions under certain conditions not tested in our standard assays. MtxA shares homology with orthologous proteins from five different organisms, two of which are not MTB. In the five organisms, the proteins are highly conserved, have not been characterized yet and no functional domain has been associated with them. The amino acid sequence alignment suggests that the conservation covers the whole protein, aside from two major exceptions: amb2230 protein (AMB-1) that lacks the N-terminal (1-63 a.a.) and Mmc1_3696 (MC-1) that has lower identity that causes the sequence alignment to be more homologous than strictly conserved. Our structure prediction of MtxA_Δ1−24_ indicates that the protein has a very diverse fold but has more β-strands than α-helices, as suggested by the CD results. MtxA_Δ1−24_ was efficiently expressed in *E. coli* and is highly soluble as monomers in solution. Additionally, the SAXS DBM demonstrates that MtxA_Δ1−24_ behaves as an extended ellipsoid with two internal domains of which one is significantly larger than the other. This can indicate that MtxA_Δ1−24_ is composed of two smaller domains connected by a flexible linker, which can explain the lack of protein crystals for the full length MtxA.

It has been shown previously that flexible proteins can be difficult to crystallize and limited proteolysis can be used to obtain crystals (Dong et al., 2007). This further supports our result that MtxA_Δ1−24_ crystals appeared only when we crystallized the protein in the presence of trypsin protease, most likely due to its cleavage. The proteolysis with trypsin yielded a smaller and stable domain that generated crystals of excellent quality, which diffracted to 2 Å resolution. We determined the crystal structure of MtxA_Δ1−141_ and compared it to the initial secondary structure prediction. Overall, the secondary structure was divided correctly into two distinct folded domains: Ig-like and TPR-like, these domains are well established and no server had predicted them in MtxA protein. When looking into the specific secondary structure elements, the secondary structure prediction is deviated from our determined structure further indicating for the inaccuracy of the prediction due to limited sequence similarity of MtxA to other proteins. The MtxA-Big domain adopts the same β-sheet arrangements, topology and conformations as Ig-like proteins, despite the low sequence identity. Furthermore, it has the same highly conserved residues as in Fn-3 protein members (Hoxha and Campion, 2014). Comparing MtxA to Tenascin-R (PDB ID code 1TDQ) indicates that they have a similar structure to Fn-3 protein members and are extracellular proteins. Tenascin-R is eukaryotic member of the tenascin family of extracellular matrix glycoproteins and although it is restricted to the nervous system and affects cell migration, adhesion and differentiation, it has no clinical consequences in knock-out animal model (Anlar and Gunel-Ozcan, 2012). The lack of phenotype is similar to the *mtxA* mutant as we were unable to discern phenotypic differences under the common standard growth conditions.

Another important structural motif of MtxA-Big is the “tyrosine corner” (Hemmingsen et al., 1994): MtxA protein has histidine instead of the tyrosine (in β-sheet F). The H201 keeps the hydrogen bond with the second a.a. in the consensus sequence (L-X-X-G-X-X-Y) and forms another hydrogen bond with the conserved D175 (in β-sheet C), whereas in members of Fn-3 family there is a hydrophobic interaction of the tyrosine with the last a.a. of β-sheet C. Additionally, all MtxA proteins contain the consensus sequence L-X-P-G-X-H (196-201 a.a.), the “histidine corner” and the highly conserved D175 and 176G in the protein. The “histidine corner” is conserved in human SOD (PDB ID code ISPD) (Deng et al., 1993) and this arrangement makes histidine corner more difficult to accommodate than tyrosine in barrel interiors (Hemmingsen et al., 1994). One of the sites that bind copper in human CuZnSOD protein is the H-V-H at the sequence L-X-X-G-X-H-X-X-H-V-H (Hart et al., 1998); since we assume that MtxA protein does not bind copper, this His corner may indicate for a binding site to other ligands or ions.

The MtxA-TPR domain has a well-known TPR motif although the TPRpred server determined that there are no significant repeats in MtxA protein. MtxA-TPR contains five anti-parallel α-helices-and-turn motifs folded as TPR, and contain the hydrophobic amino acids pattern with some of the motifsé conserved residues (D'Andrea and Regan, 2003). The consensus P32 located between the TPR motifs (Zeytuni and Zarivach, 2012) is modified to K277 in all MtxA proteins (apart from MC-1, P277). This K277 starts a highly conserved sequence in all MtxA proteins: “KDDND/E” (277-281 a.a.). This conserved “zone” exhibits a negative electrostatic potential charge at the interface between MtxA-TPR and MtxA-Big domains (Supporting Figure 3, right, top). Followed by this sequence is another highly conserved patch of MtxA, residues 282-287 “MRPLLI,” that are involved in the hydrophobic interactions between the two monomers in the asymmetric unit.

The hydrophobic patch that covers the whole face created by the TPR and the Ig-like domains may indicate for an interface for interacting with other proteins, since TPR and Ig-like folds are known to be part of protein-protein interactions (Remaut and Waksman, 2006; Zeytuni and Zarivach, 2012). In addition, the C-terminal of MtxA-TPR domain has a very negatively electrostatic potential charge caused by the following amino acids: D248, D249, D278, D279, D281, E302, E308, and E311 (a negative amino acid array). This residue composition may reveal a negatively charged concave surface in MtxA similar to the negatively charged concave surface in YrrB (PDB ID code 2Q7F) (Han et al., 2007).

Since the full MtxA structure, based on our SAXS model, displays an elongated structure divided into large and small connected ellipsoids, we believe that the large ellipsoid is the MtxA_Δ1−141_ and small ellipsoid is probably the missing MtxA_25−141_ domain. Since our results did not implicate MtxA function and since we missed a full domain, we wanted to obtain its full structure as a means to obtain a predicted function. For that, we submitted MtxA to the CASP competition (Critical Assessment of Techniques for Protein Structure Prediction) (Kryshtafovych et al., 2014). From the CASP results (T0828) we found two “close” predictions out of the full structure list, the first from nns_TS1 group and the second from QUARK_TS2 group. Structural superimpositions of MtxA_Δ1−141_ with nns_TS1 and QUARK_TS2 resulted in structural superposition with RMS deviation of 1.54 Å and 1.40 Å, with 58 and 72 superposed Cα atoms at the MtxA-Big and MtxA-TPR fold, respectively. This signifies that the MtxA protein has a unique fold and may have an important role in signaling at the periplasm.

In summary, we have applied a wide range of methodologies in order to characterize MtxA_Δ1−24_ in solution. We found that MtxA is a monomeric, 34.7 kDa protein with an extended ellipsoid shape containing at least two internal domains. We have determined the MtxA_Δ1−141_ structure and disclosed its unique fold. The MtxA protein probably has an importance in periplasmic or extracellular interactions that could act as a sensor or mediating interaction with other proteins or even activating signals in the bacteria.

### Conflict of interest statement

The authors declare that the research was conducted in the absence of any commercial or financial relationships that could be construed as a potential conflict of interest.
