# Supplementary material for: Crystal structure of the magnetobacterial protein MtxA C-terminal domain reveals a new sequence-structure relationship
Source: Front Mol Biosci. 2015 May 21;2:25. doi: 10.3389/fmolb.2015.00025 (PMC4439547; doi:10.3389/fmolb.2015.00025)
Supplement: Supplementary file 1 [file DataSheet1.DOCX]

**Supporting Information: Crystal structure of the magnetobacterial protein MtxA C-terminal domain reveals a new sequence-structure relationship**

**Geula Davidov^1^, Frank D. Müller^2^, Jens Baumgartner^3^,** **Ronit Bitton^4^, Damien Faivre ^3^, Dirk Schüler^2^, and Raz Zarivach^1^**

^1^Department of Life Sciences and the National Institute for Biotechnology in the Negev, Ben-Gurion University of the Negev, Beer Sheva, Israel

^2^Department of Microbiology, University of Bayreuth, Bayreuth, Germany

^3^Department of Biomaterials, Max Planck Institute of Colloids and Interfaces, Science Park Golm, 14424 Potsdam, Germany

^4^Ilse Katz Institute for Nanoscale Science and Technology and Department of Chemical Engineering, Ben Gurion University of the Negev, Beer-Sheva, Israel

^#^Correspondence should be addressed to Raz Zarivach, Department of Life Sciences, Ben Gurion University of the Negev, P.O.B. 653, Beer-Sheva 84105, Israel. Tel: +972-8-6461999, Fax: +972-8-6472970, Email: [zarivach@bgu.ac.il](mailto:zarivach@bgu.ac.il)

**Supporting Figure 1: Circular dichroism analysis of MtxA_∆1-24_**

(A) Dichroic spectra for MtxA_∆1-24_. (B) Melting curve of MtxA_∆1-24_, measured at 222 nm (blue line indicates calculated *T*_m_).

**Supporting Figure 2: MtxA_∆1-24_ MALDI-TOF analysis**

Matrix-assisted laser desorption/ionization (MALDI) time-of-flight (TOF) mass spectra of (A) MtxA_∆1-24_ concentrated to 15 mg/ml. (B) MtxA_∆1-24_ after trypsin treatment in a ratio of 1:4000.

**Supporting Figure 3:** **Sequence alignment of MtxA-Big and MtxA-TPR domains**

(A) Amino acid sequence alignment of MtxA-Big with fibronectin EDA (PDB ID code 1J8K), Tenascin-R (PDB ID code 1TDQ-A), and Fibronectin first type III module (PDB ID code 1OWW).

(B) Amino acid sequence alignment of MtxA-TPR with the synthetic consensus TPR protein (PDB ID code 2AVP-A), MamAR50E (PDB ID code 3ASD-A), and YrrB protein (PDB ID code 2Q7F-B).

The output is from the ClustalW2 server; http://www.ebi.ac.uk/Tools/msa/clustalw2 and ESPript 3.0; http://espript.ibcp.fr/ESPript/cgi-bin/ESPript.cgi. Strictly conserved residues are highlighted with a red background and highly homologous residues are boxed.

**Supporting Figure 4: Surface charge representations of MtxA_Δ1-141_ protein**

Surface charge representations of MtxA_∆1-141_, with blue and red colors representing regions of positive and negative electrostatic potential, respectively, and the overall position of the structure in ribbon. The molecule is shown in four views, related by 180° and 90° rotations. (Top) The overall protein with two views (left and right). (Left, bottom) The surface displays an extreme negative charge distribution. (Right, bottom) The surface displays the electrostatic charge distribution. All electrostatic surfaces representations were produced with the APBS plug-in of PyMOL.

**Supporting Figure 5: Polar interaction network between the two monomers in the asymmetric unit**

(A) The MtxA_Δ1-141_ crystal contains two protein monomers in the asymmetric unit. The two monomers of MtxA_Δ1-141_ are: MtxA chain A – L136-S311, and MtxA chain B – K137-E305. The molecules are shown in a 90° rotation view. (B) The MtxA_Δ1-141_ structure colored according to their B-factor (deep blue-low B-factor; yellow-high B-factor). (C) ‘Close up’ view of the interacting charged amino acids between MtxA-TPR chain-A K294 to MtxA-Big chain-B D149. Blue dotted lines indicate for contact distances. (D) ‘Close up’ view of the MtxA-Big fold hydrogen-bond interaction network (blue dotted lines) between side chains (shown as sticks) and water molecules (shown as red spheres). MtxA-Big chain-A represented as orange cartoon and MtxA-Big chain-B represented as brown cartoon. (E) Overview of the MtxA-TPR fold hydrogen-bond interaction network (red dotted lines) between the two chains of the MtxA-TPR fold. Side chains are shown as sticks and water molecules are shown as red spheres.

**Supporting Figure 6: Transmission electron microscopy (TEM) images of *mtxA* mutant cells**

(A) Image depicting three individual cells, each containing a wild type-like magnetosome chain (indicated by black arrows). (B) Magnified section of the sample in (A) suggesting that the magnetite crystals are of normal shape and size.

**Supporting Figure 7: Swim ring formation of wild type and *mtxA* mutant**

Swim ring formation of wild type (upper wells) and *mtxA* mutant (lower wells) in 0.2% motility agar and in the presence of a magnetic field (N-S). The distortion of the swim ring shape indicates that cell movement in both strains is similarly biased by the field direction. Five microliters of overnight culture was pipetted into the swim agar and plates were incubated under microoxic conditions for two days, as described by Popp *et al*., 2014.

**Supplementary Figure 8: An electron density map of MtxA_Δ1-141_**

A represented sigma-A weighted 2Fo-Fc electron density map in grey (1.5σ) around MtxA_Δ1-141_ (shown in sticks). Water molecules are shown as red spheres.

**Supporting Table 1: Composition of crystallization buffers that resulted in crystal hits for MtxA_∆1-24_ (Native and SeMet)**

| **MtxA** | **Screen** | **Condition No.** | **Salt** | **Buffer** | **Precipitant** | **Crystal notes** |
| --- | --- | --- | --- | --- | --- | --- |
| SeMet | Index | 2 |  | 0.1 M Sodium acetate trihydrate pH 4.5 | 2.0 M Ammonium sulfate | Clusters of tiny needle crystals |
| SeMet | Index | 39 |  | 0.1 M HEPES pH 7.0 | 30% v/v Jeffamine ® ED-2001 pH 7.0 | Clusters of tiny needle crystals |
| Native, SeMet | Index | 42 |  | 0.1 M Bis-tris pH 5.5 | 25% PEG 3,350 | Clusters of tiny needle crystals |
| SeMet | Index | 44 |  | 0.1 M HEPES pH 7.5 | 25% PEG 3,350 | Clusters of tiny needle crystals |
| Native, SeMet | Index | 70 | 0.2 M Sodium chloride | 0.1 M Bis-tris pH 5.5 | 25% PEG 3,350 | Clusters of tiny needle crystals |
| SeMet | Index | 79 | 0.2 M Ammonium acetate | 0.1 M Bis-tris pH 6.5 | 25% PEG 3,350 | Clusters of tiny needle crystals |
| SeMet | Index | 81 | 0.2 M Ammonium acetate | 0.1 M Tris pH 8.5 | 25% PEG 3,350 | Clusters of tiny needle crystals |
| Native, SeMet | Index | 85 | 0.2 M Magnesium chloride hexahydrate | 0.1 M Tris pH 8.5 | 25% PEG 3,350 | Clusters of tiny needle crystals |
| SeMet | Index | 87 |  | 0.2 M Sodium malonate pH 7.0 | 20% PEG 3,350 | Clusters of tiny needle crystals |
| Native, SeMet | Index | 93 | 0.05 M Zinc acetate dihydrate |  | 20% PEG 3,350 | Clusters of tiny needle crystals |

**Supporting Table 2: RMSD of superimpositions and sequence identity between MtxA-Big and MtxA-TPR to other proteins in the PDB**

| **MtxA domain** | **PDB code** | **RMS (**Å) | **No. of atom (**Cα) | **Seq. Identity (%)** | **Seq. similarity (%)** | **Dali score (Z value)** |
| --- | --- | --- | --- | --- | --- | --- |
| MtxA-Big | 1DTQ | 1.40 | 62 | 7.36 | 15.29 | 8.3 |
|  | 1J8K | 1.57 | 55 | 5.31 | 11.76 | 8.4 |
|  | 1OWW | 1.46 | 56 | 3.22 | 8.23 | 7.4 |
| MtxA-TPR | 2AVP | 1.20 | 48 | 4.41 | 11.49 | 10.4 |
|  | 3ASD | 1.39 | 57 | 7.36 | 12.64 | 10.4 |
|  | 2Q7F | 1.39 | 63 | 3.77 | 8.04 | 11.3 |

RMSD of superimpositions and sequence identity between MtxA-Big and MtxA-TPR domains with some of the Dali server-fitted structures. The average RMS and the number of common Cα atoms output were generated by Swiss-PdbViewer. The output of sequence identity and similarity was produced in the SIAS server; http://imed.med.ucm.es/Tools/sias.html.
