# Supplementary figures and images for: Crystal structure of the magnetobacterial protein MtxA C-terminal domain reveals a new sequence-structure relationship
Source: Front Mol Biosci. 2015 May 21;2:25. doi: 10.3389/fmolb.2015.00025 (PMC4439547; doi:10.3389/fmolb.2015.00025)

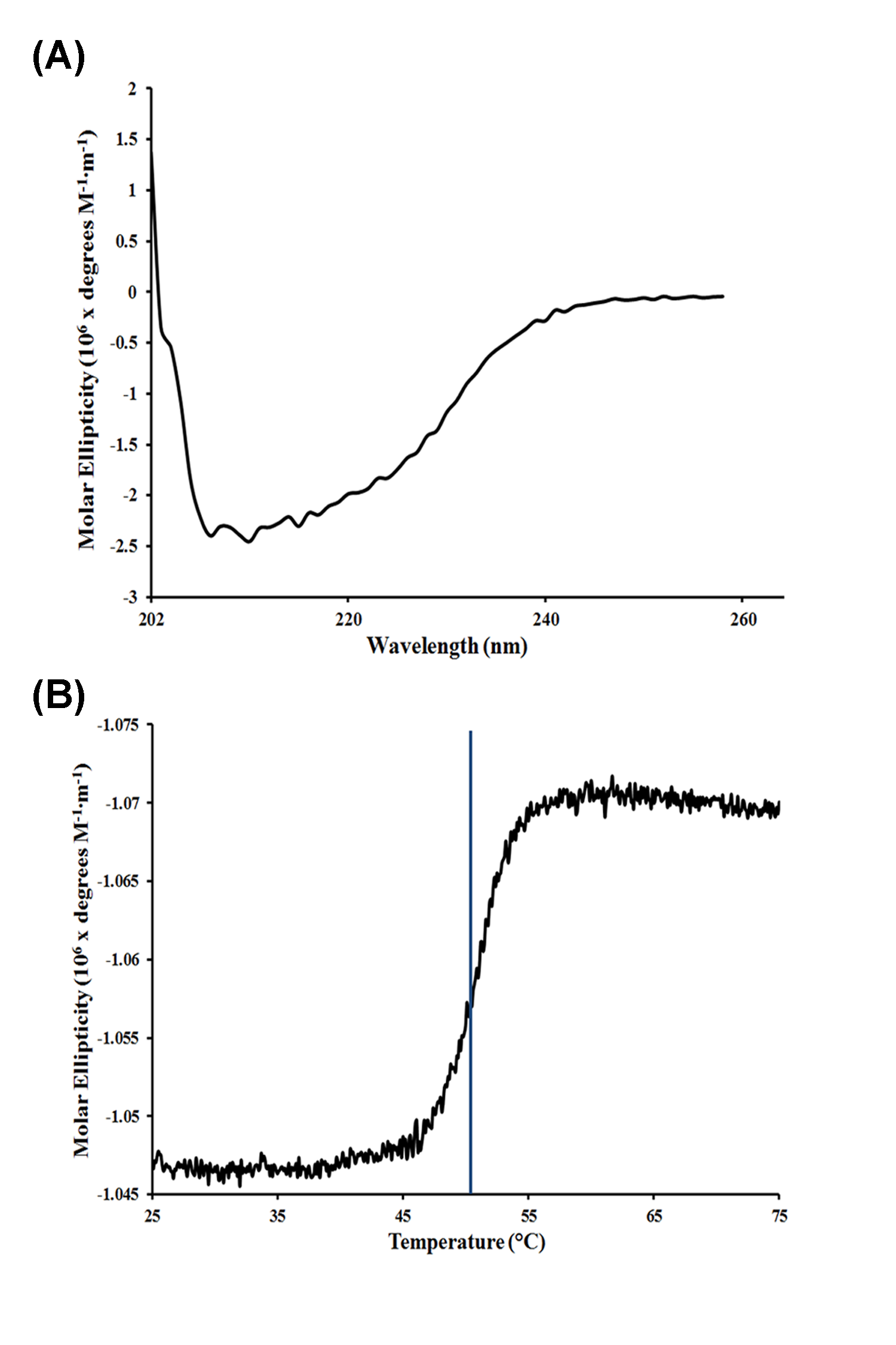

Supplement: Supplementary file 2 [file Image1.TIF]

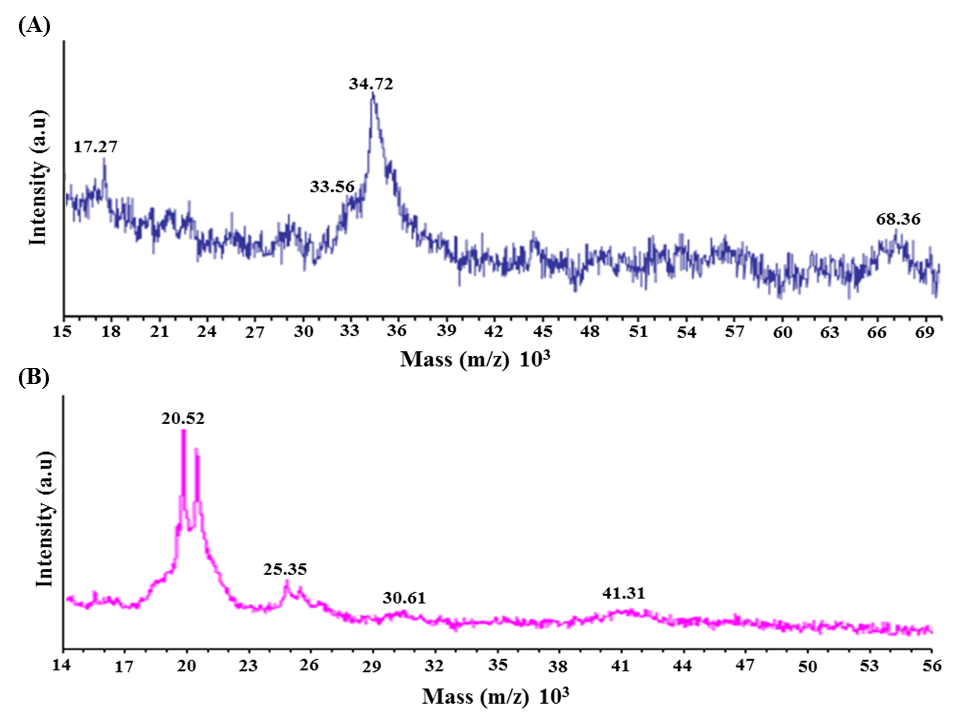

Supplement: Supplementary file 3 [file Image2.TIF]

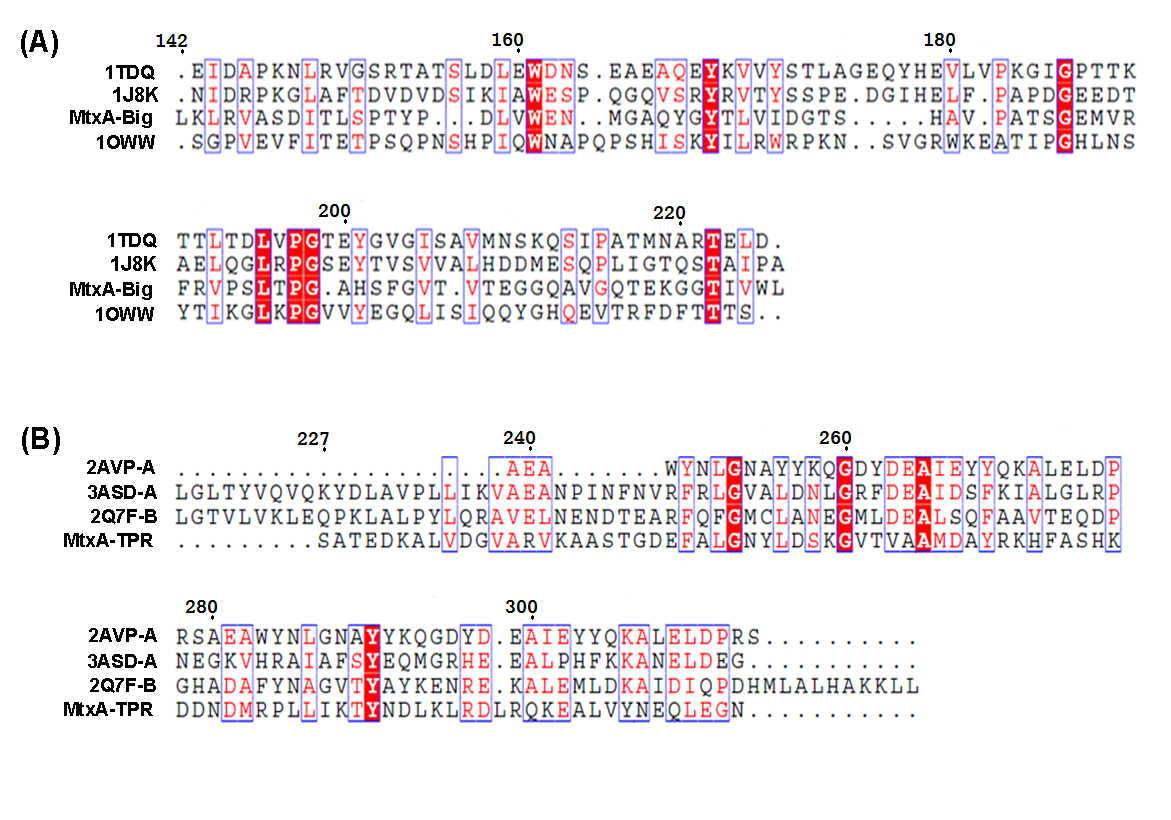

Supplement: Supplementary file 4 [file Image3.TIF]

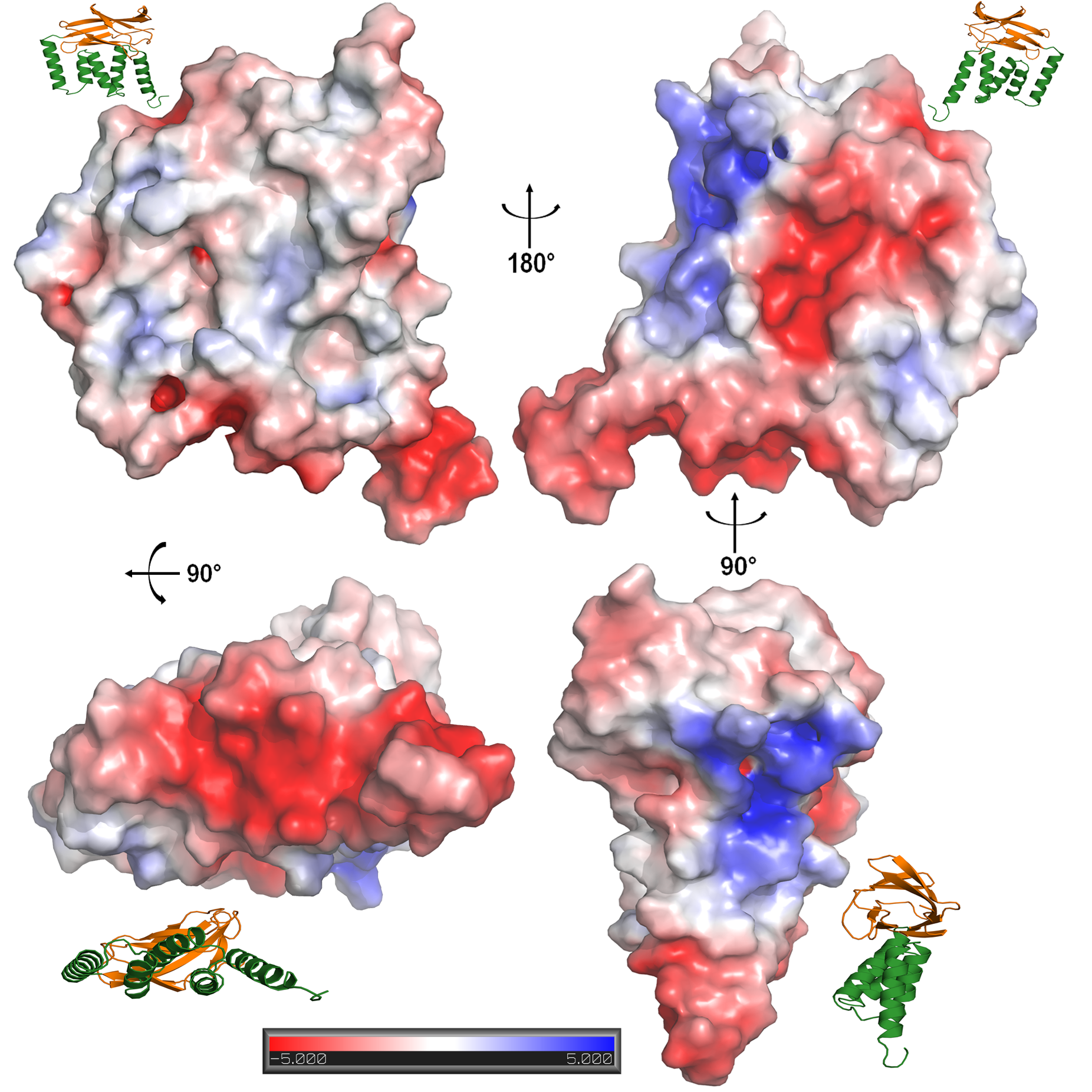

Supplement: Supplementary file 5 [file Image4.TIF]

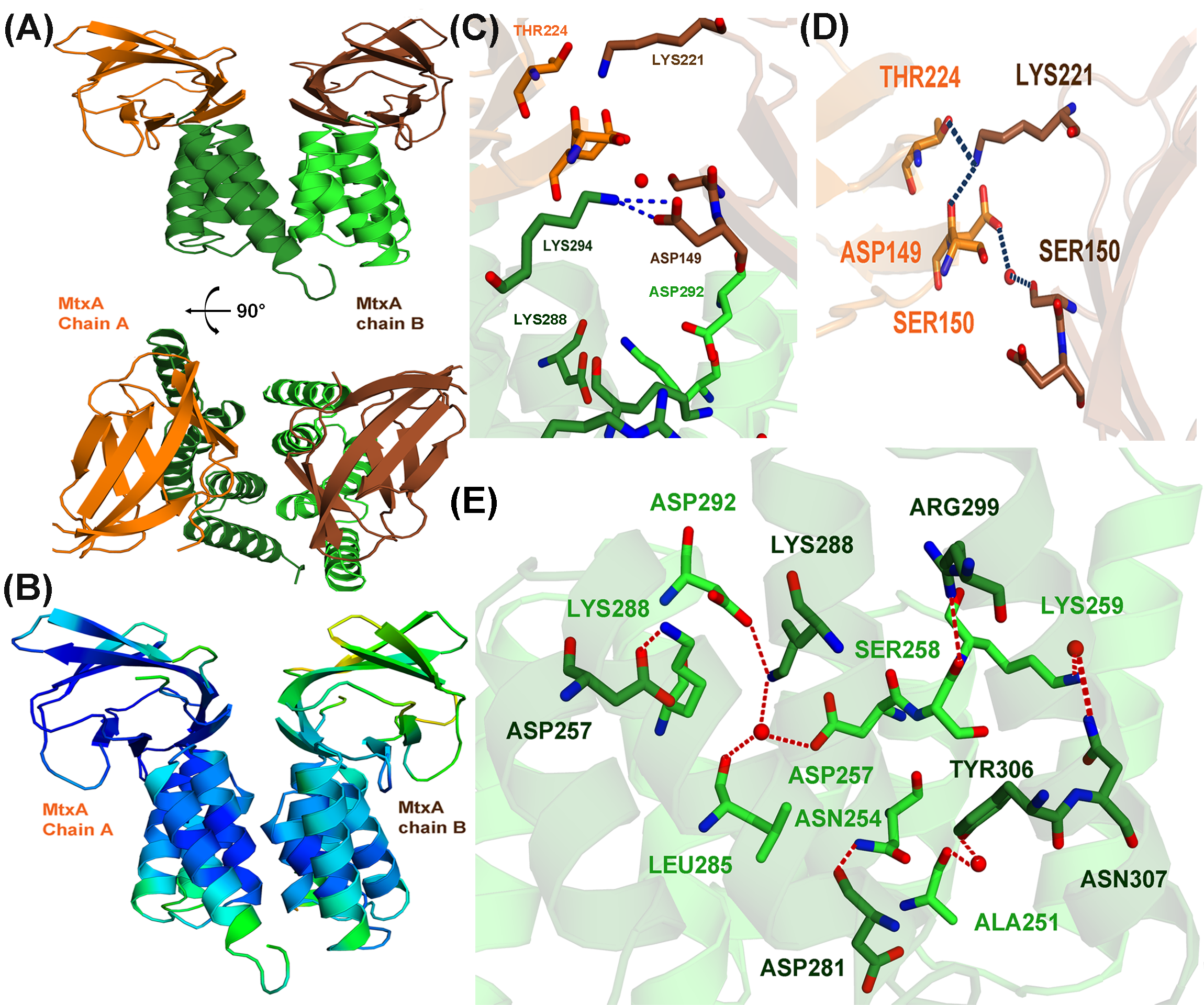

Supplement: Supplementary file 6 [file Image5.TIFF]

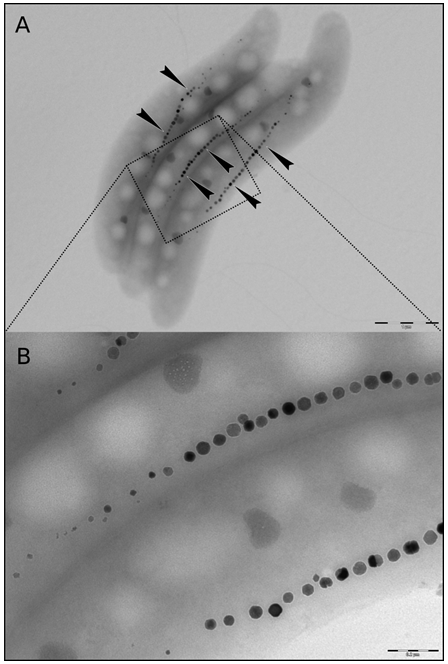

Supplement: Supplementary file 7 [file Image6.TIF]

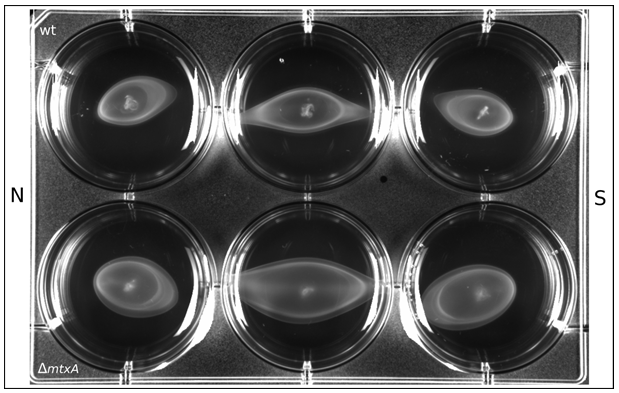

Supplement: Supplementary file 8 [file Image7.TIF]

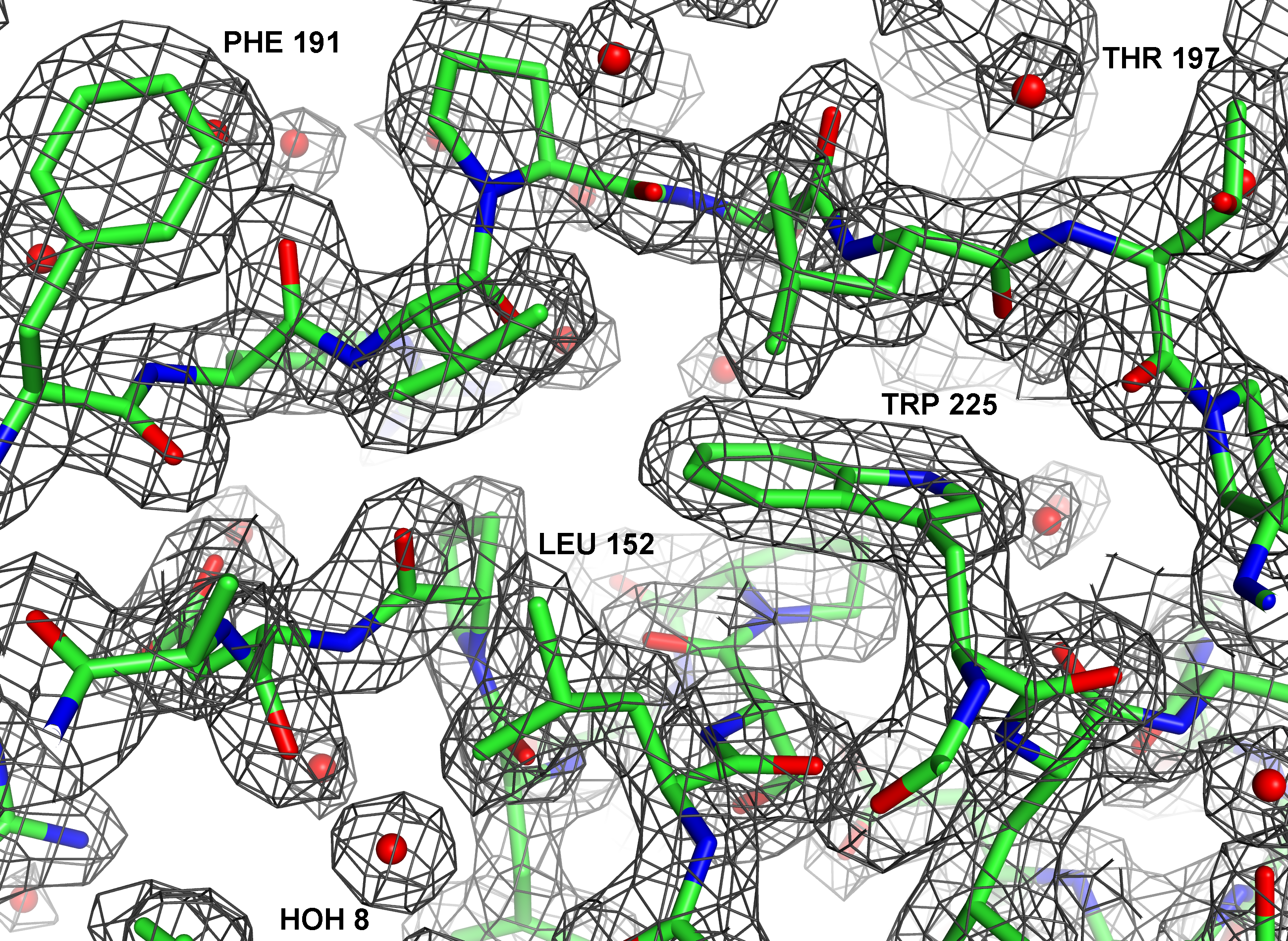

Supplement: Supplementary file 9 [file Image8.TIF]
